# Diagnostic accuracy of the ROSIER scale for detecting acute ischemic stroke: a comparison with FAST-ED, CPSS, and reference criteria

**DOI:** 10.1186/s12873-026-01654-0

**Published:** 2026-06-30

**Authors:** Farhad Heydari, Mobina Marzban, Mehrdad Esmailian, Amir Bahador Boroumand, Afshin Najafi, Mehdi Nasr Isfahani, Elahe Nasri Nasrabadi, Ali Emamjomeh

**Affiliations:** 1 Department of Emergency Medicine, Faculty of Medicine, Isfahan University of Medical Sciences, Isfahan, Iran; 2 Department of Neurology, School of Medicine, Isfahan University of Medical Sciences, Isfahan, Iran; 3 Isfahan Cardiovascular Research Center, Cardiovascular Research Institute, Isfahan University of Medical Science, Isfahan, Iran

**Keywords:** Acute ischemic stroke, Emergency department, ROSIER, CPSS, FAST-ED, NIHSS

## Abstract

**Background:**

Acute ischemic stroke (AIS) requires rapid diagnosis and treatment. While neuroimaging remains the gold standard, it is often unavailable in early emergency or prehospital settings. Clinical stroke scales may bridge this gap. Thus, this study aimed to compare the diagnostic accuracy of the ROSIER scale with the Cincinnati Prehospital Stroke Scale (CPSS), Field Assessment Stroke Triage for Emergency Destination (FAST-ED), and National Institutes of Health Stroke Scale (NIHSS) for the detection of AIS in patients with suspected stroke, using MRI as the imaging reference standard.

**Methods:**

This prospective cross-sectional study was performed on adult patients admitted to the emergency department (ED) between June 2021 and 2023, with acute neurological symptoms and underwent brain MRI for a suspected stroke. After data gathering, receiver operating characteristic (ROC) curve analysis was performed, and sensitivity, specificity, positive predictive value, and negative predictive value of ROSIER for detecting of AIS were calculated and compared with CPSS, FAST-ED, and NIHSS. MRI was considered the gold standard for the diagnosis of AIS.

**Results:**

A total of 655 patients were included, and 477 (72.8%) were diagnosed with AIS. The mean age was 68.43 ± 13.01 years, and 57.3% were male. The AUROC values for ROSIER, CPSS, FAST-ED, and NIHSS were 0.813, 0.860, 0.811, and 0.822, respectively. No significant differences were found between the tools. Sensitivities for ROSIER ≥ 2, CPSS ≥ 2, FAST-ED ≥ 3, and NIHSS ≥ 8 were 86.8%, 90.7%, 74.7%, and 73.5%, respectively. Their specificities were 58.1%, 67.7%, 77.4%, and 81.5%, respectively.

**Conclusion:**

All four tools showed good diagnostic accuracy. CPSS was the most sensitive and suitable for prehospital detection, whereas NIHSS offered the best specificity for in-hospital confirmation. Selection of the tool should be context-dependent, based on resources, time, and examiner expertise.

## Introduction

Stroke is a serious public health issue and remains the leading cause of death and long-term disability globally. The Global Burden of Disease Study (GBD) reports that in 2021, there were nearly 12.2 million new cases of stroke and 7.8 million stroke-related deaths worldwide [1]. Among the many strokes, acute ischemic stroke (AIS) is the most prevalent which impedes cerebral perfusion and triggers rapid neuronal damage [2]. Early detection and treatment of AIS are crucial due to the urgency of therapeutic interventions [2].

Advanced neuroimaging modalities, including computed tomography angiography (CTA) and magnetic resonance imaging (MRI), are reference standard tools for the diagnosis of AIS and for guiding therapeutic interventions [3]. However, these modalities are often unavailable in prehospital or early emergency settings, posing challenges to the timely diagnosis of AIS [4]. Therefore, several stroke assessment tools have been developed to enhance early diagnosis and ensure appropriate triage of stroke patients to specialized stroke centers [5].

Among the many tools available, the Recognition of Stroke in the Emergency Room (ROSIER) scale has been proven to distinguish between strokes and non-stroke entities such as seizures, syncope, and hypoglycemia [6]. The Field Assessment Stroke Triage for Emergency Destination (FAST-ED) and the Cincinnati Prehospital Stroke Scale (CPSS) are also widely used bedside assessments designed to support rapid identification of suspected stroke in emergency and prehospital settings [7, 8]. Another important tool in the acute stroke setting is the National Institutes of Health Stroke Scale (NIHSS), which is used regularly to measure stroke severity and determine treatment planning [9, 10]. In addition, most previous research has used clinical diagnosis as a reference standard rather than more definitive neuroimaging methods like CT or MR angiography, which may limit the validity of diagnostic accuracy estimates for AIS detection [7, 11].

Thus, this study aimed to compare the diagnostic accuracy of the ROSIER scale with CPSS, FAST-ED, and NIHSS for the detection of AIS in patients with suspected stroke, using MRI as the imaging reference standard.

## Method and materials

### Study design

This prospective cross-sectional study was performed between June 2021 and 2023 in the emergency departments (ED) of Al-Zahra and Ayatollah Kashani Hospitals, Isfahan, Iran. The hospitals are the main regional referral centers for neurological emergencies. The main aim of the study was to assess the ROSIER scale for the detection of AIS. We also compared the diagnostic accuracy of the ROSIER scale with that of the FAST-ED and CPSS, using the final clinical diagnosis and neuroimaging findings as reference standard.

The inclusion criteria were adult patients 18 years and older who presented to the ED with acute neurological symptoms suggestive of stroke, such as acute hemiparesis, facial droop, slurred speech, or impaired coordination, and who had an MRI as part of their diagnostic evaluation. The exclusion criteria were patients with known intracranial hemorrhage on imaging, patients transferred from other medical centers, patients who discharged themselves before a definitive diagnosis was made, or patients whose consciousness level prevented accurate assessment using the stroke evaluation tools. This study was conducted in accordance with the ethical principles of the Declaration of Helsinki. The study protocol was approved by the Ethics Committee of Isfahan University of Medical Sciences (IR.MUI.MED.REC.1402.368). Informed consent was obtained from all subjects or their legal guardians before participation.

### Variables and data measurement

The ROSIER scale is composed of seven clinical features, such as facial weakness, arm weakness, leg weakness, speech disturbance, visual field defect, seizure activity, and loss of consciousness. The score ranges from − 2 to + 5, and a score of 2 or higher is positive for stroke [12]. The FAST-ED scale comprises five elements—facial palsy, arm weakness, speech disturbance, eye deviation, and neglect—with scores from 0 to 9; a score of 3 or more is suggestive of high risk of AIS [7]. The CPSS assesses three clinical signs: facial droop, arm drift, and speech abnormalities. Each item is scored dichotomously, and the total score ranges from 0 to 3, with a score of 2 or more indicating a positive screen [8]. The NIHSS is an extensive tool used to measure the severity of neurological impairment in acute stroke, and a score of 8 or more was deemed to indicate moderate to severe stroke [13].

The reference standard for the final diagnosis AIS was the adjudicated diagnosis based on the clinical presentation and neuroimaging findings [14]. The imaging was done with MRI.

Other variables were demographic factors and vascular risk factors that could affect both the presentation of stroke and diagnostic accuracy. These were age, sex, and clinical history of hypertension, diabetes mellitus (DM), ischemic heart disease (IHD), atrial fibrillation (AF), previous stroke or transient ischemic attack (TIA), and smoking status. These were included to fully describe the study population and determine possible confounders in the diagnostic performance of the stroke scales being assessed.

All patients fulfilling the inclusion criteria were recruited on a consecutive sampling basis to minimize selection bias. All stroke scale evaluations were performed by trained emergency medicine doctors on immediate patient arrival to the emergency department, before neuroimaging. The ROSIER, FAST-ED, and CPSS instruments were used independently and in sequence, with each score being documented before any diagnostic imaging or neurologist assessment to prevent observer bias. The on-call stroke neurologist then completed the NIHSS as part of the initial formal neurological examination.

Following clinical assessment, all patients underwent an MRI. Imaging was interpreted independently by two board-certified neuroradiologists, both blinded to the clinical assessment scores and final diagnosis. In cases of disagreement, a consensus reading or evaluation by a third expert was obtained.

To ensure that the methods were robust, we had rigorous blinding procedures. The clinicians who performed the stroke scale measurements were unaware of the imaging findings or final diagnosis. The radiologists were only able to view the patient’s age and the time of symptom onset. A stroke neurologist determined the final diagnosis after reviewing the patient’s clinical presentation, NIHSS score, and MRI findings. The neurologist was unaware of the stroke scale scores in the prehospital and emergency department.

### Sample sizes

Sample size was estimated a priori using Buderer’s method for diagnostic accuracy studies [15]. The calculation was based on an assumed sensitivity of 92% and specificity of 86% for the ROSIER scale [16], and an expected AIS prevalence of 69.9% among suspected stroke presentations [17]. Using a two-sided 95% confidence level (Z = 1.96) and an absolute precision of 5% (d = 0.05), the required sample size was calculated as 615 patients, with specificity determining the larger sample requirement. Although the minimum required sample size was 615, we ultimately enrolled 655 consecutive patients. Including all eligible patients increased statistical power and reduced the risk of selection bias without altering the original effect-size assumptions.

### Statistical analysis

All statistical analyses were performed using SPSS version 25 (IBM Corp., Armonk, NY, USA). A two-tailed p-value of less than 0.05 was considered statistically significant. Descriptive statistics were used to define the demographic and clinical profile of the study population. Continuous variables, such as age, blood glucose, and duration from onset of symptoms to presentation in the emergency department, were presented as means ± standard deviation (SD) and compared using the independent samples t-test. For categorical variables like sex, medical history (hypertension, DM, IHD, AF, and smoking history), and occurrence of seizures, comparisons were made using either the Chi-square test or Fisher’s exact test as appropriate. Receiver operating characteristic (ROC) curve analyses were carried out to determine the diagnostic accuracy of the four clinical scales—ROSIER, FAST-ED, CPSS, and NIHSS—in the prediction of AIS. Area under the curve (AUC) was determined for each scale, along with the corresponding 95% confidence intervals (CI). Multiple diagnostic metrics, including sensitivity, specificity, positive predictive value (PPV), negative predictive value (NPV), positive likelihood ratio (PLR), and negative likelihood ratio (NLR), were calculated at set optimal cut-off scores (ROSIER ≥ 2, FAST-ED ≥ 3, CPSS ≥ 2, and NIHSS ≥ 8). The Youden index for each scale was determined by finding the optimal trade-off between sensitivity and specificity. Comparisons of AUCs between the tools were carried out using the DeLong method, a non-parametric method for comparison of correlated ROC curves.

## Results

**Fig. 1:**
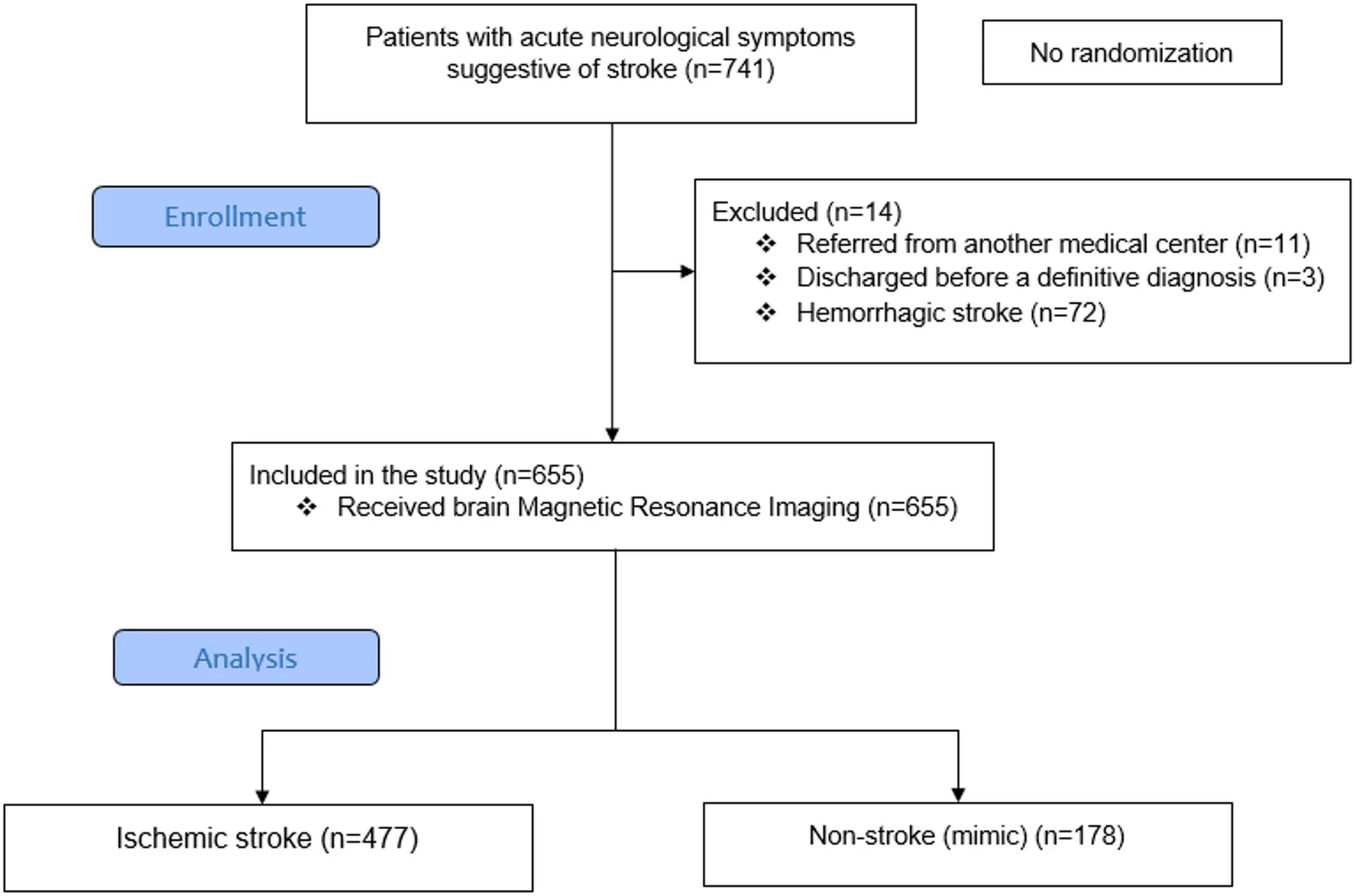
CONSORT flow diagram of patient selection, exclusions, and diagnostic classification

The final analysis contained 655 patients. 477 of these (72.8%) experienced an AIS (Fig. 1). The mean age of the stroke patients was 68.43 ± 13.01 years, which was higher than that of the non-stroke subjects (64.34 ± 13.82; *P* < 0.001). The stroke group also had a higher proportion of men (59.7%; *P* = 0.041). Other clinical and historical variables, including hypertension, DM, smoking, IHD, coagulopathy, previous stroke, hyperlipidemia, and seizures, were not different between the groups (Table 1).

**Table 1:** Demographic characteristics and history of disease and risk factors in patients with and without stroke diagnosis

	Stroke (n=477)	Non-stroke (n=178)	P-value
***Age; mean (SD), year***	68.43 ± 13.01	64.34 ± 13.82	<0.001
***Gender***			
*** Male***	285 (59.7)	90 (50.6)	0.041
*** Female***	192 (40.3)	88 (49.4)	
***Blood sugar level (mg/dL), mean (SD)***	149.39 ± 63.68	144.82 ± 75.79	0.439
***Symptom onset to ED arrival, mean (SD), hour***	4.32 ± 6.94	12.05 ± 10.02	<0.001
***History of hypertension, n (%)***			
*** Negative***	190 (39.8)	59 (33.1)	0.125
*** Positive***	287 (60.2)	119 (66.9)	
***History of IHD, n (%)***			
*** Negative***	294 (61.6)	122 (68.5)	0.121
*** Positive***	183 (38.4)	56 (31.5)	
***History of smoking, n (%)***			
*** Non-smoker***	400 (83.9)	146 (82.0)	0.558
*** Smoker***	77 (16.1)	32 (18.0)	
***DM, n (%)***			
*** Negative***	336 (70.4)	120 (67.4)	0.504
*** Positive***	141 (29.6)	58 (32.6)	
***Coagulopathy, n (%)***			
*** Negative***	468 (98.1)	170 (95.5)	0.093
*** Positive***	9 (1.9)	8 (4.5)	
***History of stroke, n (%)***			
*** Negative***	417 (87.4)	148 (83.1)	0.162
*** Positive***	60 (12.6)	30 (16.9)	
***HLP, n (%)***			
*** Negative***	394 (82.6)	140 (78.7)	0.259
*** Positive***	83 (17.4)	38 (21.3)	
***Seizure, n (%)***			
*** Negative***	470(98.5)	172 (96.6)	0.128
*** Positive***	7 (1.5)	6 (3.4)	

Derived from independent t-tests and chi-square tests for continuous and categorical variables, respectively

Values are presented as mean ± standard deviation (SD) for continuous variables and as frequency (percentage) for categorical variables

Abbreviations: ED, emergency department; IHD, ischemic heart disease; DM, diabetes mellitus; HLP, hyperlipidemia

Table 2 presents the diagnostic performance of four stroke assessment tools: FAST-ED, CPSS, ROSIER, and NIHSS. CPSS had the highest sensitivity of 90.7% (95% CI: 87.4–93.4) with a cut-off ≥ 2. This was followed by ROSIER by 86.8% (95% CI: 82.4–90.5), while NIHSS had the highest specificity (81.48%, 95% CI: 61.9–93.7), which means it is better at ruling out non-stroke patients. For the measure of PPV, CPSS achieved the highest of 87.2% (95% CI: 85.5–88.3). NIHSS achieved the highest NPV value of 63.0% (95% CI: 42.4–80.6). For the likelihood ratio, NIHSS achieved the highest PLR value of 3.97 (95% CI: 1.79–8.78). CPSS had the smallest value of NLR at 0.14 (95% CI: 0.09–0.20), which means CPSS is strong in excluding the disease when the test is non-positive.

**Table 2:** The ROC analysis results of four different scores in prediction of acute ischemic stroke

Variables	NIHSS	ROSIER	CPSS	FAST-ED
**Cut-off**	≥ 8	≥ 2	≥ 2	≥ 3
**Sensitivity(95% CI)**	73.5 (68.0–78.5)	86.8 (82.4–90.5)	90.7 (87.4–93.4)	74.7 (69.3–79.5)
**Specificity(95% CI)**	81.5 (61.9–93.7)	58.1 (39.1–75.5)	67.7 (48.6–83.3)	77.4 (58.9–90.4)
**PPV(95% CI)**	86.7 (82.3–90.5)	81.5 (61.9–93.7)	87.2 (85.5–88.3)	86.9 (84.3–88.4)
**NPV(95% CI)**	63.0 (42.4–80.6)	51.8 (31.9–71.3)	48.8 (42.2–54.0)	37.6 (32.0–43.5)
**PLR(95% CI)**	3.97 (1.79–8.78)	3.07 (1.59–5.91)	2.81 (1.69–4.69)	3.31 (1.72–6.37)
**NLR(95% CI)**	0.32 (0.25–0.42)	0.40 (0.31–0.51)	0.14 (0.09–0.20)	0.33 (0.25–0.43)
**AUC(95% CI)**	0.822 (0.776–0.862)	0.813 (0.766–0.854)	0.860 (0.818–0.896)	0.811 (0.764–0.852)
**Youden index**	0.550 (0.398–0.661)	0.467 (0.294–0.584)	0.584 (0.429–0.729)	0.521 (0.365–0.654)
**P value**	< 0.001	< 0.001	< 0.001	< 0.001

Derived from ROC curve analysis. Values represent sensitivity, specificity, positive predictive value (PPV), negative predictive value (NPV), positive likelihood ratio (PLR), negative likelihood ratio (NLR), area under the ROC curve (AUC), and Youden index for each tool at its pre-specified cutoff

Abbreviations: AUC, area under the curve; PPV, positive predictive value; NPV, negative predictive value; PLR, positive likelihood ratio; NLR, negative likelihood ratio; CI, confidence interval

**Fig. 2:**
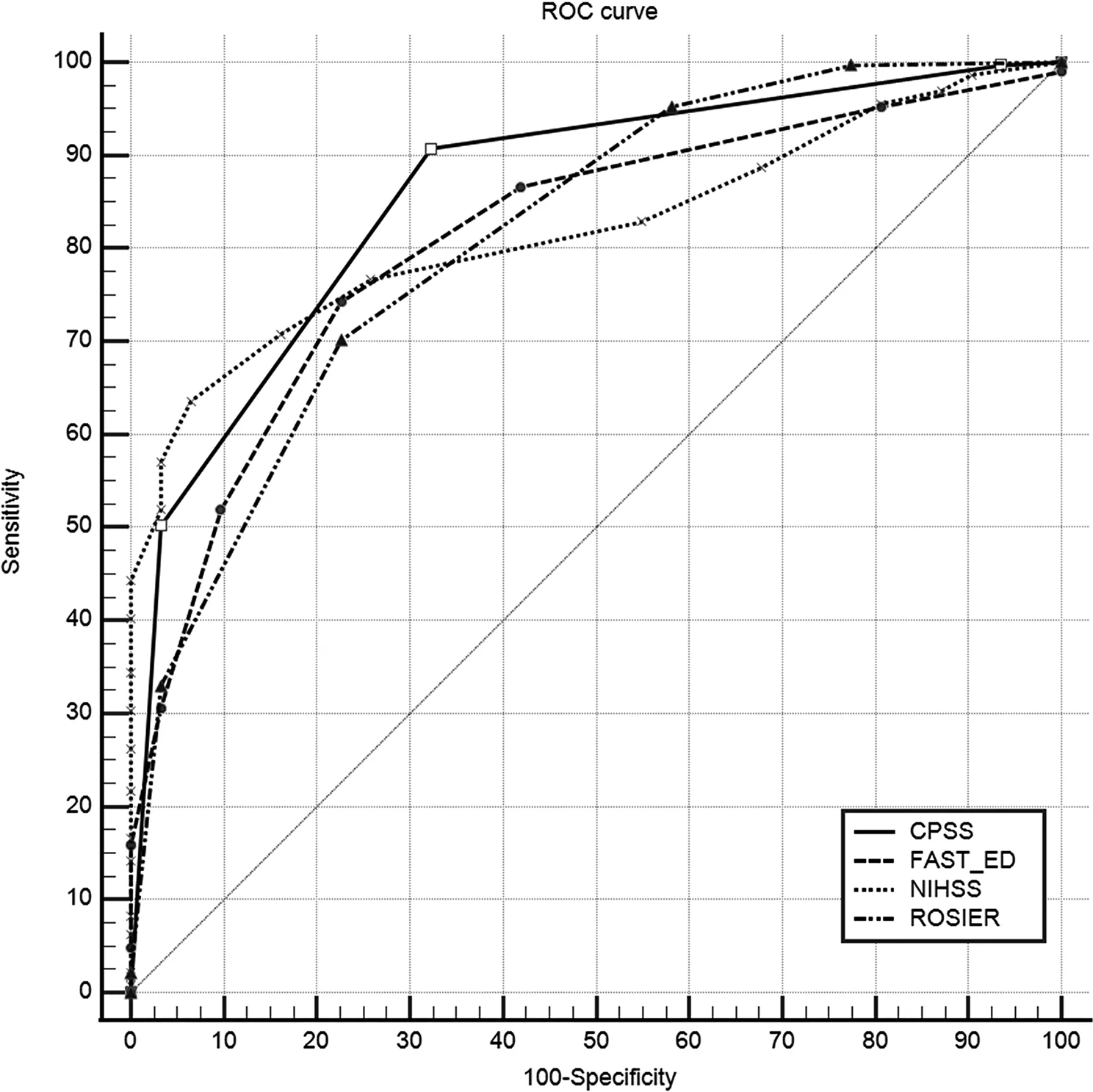
Receiver operating characteristic (ROC) curves for the CPSS, FAST-ED, ROSIER, and NIHSS tools in predicting acute ischemic stroke (AIS)

The area under the ROC curves (AUROC) of ROSIER, CPSS, FAST-ED, and NIHSS score for predicting AIS were 0.813, 0.860, 0.811, and 0.822, respectively (Table 2; Fig. 2).

Table 3 presents the comparison of the AUROC of diagnostic tools by their p-values. The findings indicate there are no significant differences in their performances. ROSIER was similar to CPSS, FAST-ED, and NIHSS in predicting AIS.

**Table 3:** Comparison of the different AUROC of four different tools with statistical significance (p value)

	NIHSS	ROSIER	CPSS	FAST-ED
NIHSS		0.827	0.144	0.793
ROSIER			0.275	0.969
CPSS				0.257
FAST-ED				

Table 1 Derived from DeLong’s non-parametric test for comparing correlated ROC curves.Values represent p-values for differences in area under the ROC curves (AUROC) between each pair of stroke tools

Abbreviations: AUROC, area under the receiver operating characteristic curve; CPSS, Cincinnati Prehospital Stroke Scale; FAST-ED, Field Assessment Stroke Triage for Emergency Destination; ROSIER, Recognition of Stroke in the Emergency Room; NIHSS, National Institutes of Health Stroke Scale

## Discussion

This research compared the diagnostic accuracy of four stroke scales—CPSS, FAST-ED, ROSIER, and NIHSS— in the emergency setting, using the final diagnosis based on clinical evaluation and MRI findings as the reference standard. Overall, CPSS emerged as the most sensitive tool and performed best for early detection, whereas NIHSS showed greater specificity and a stronger ability to rule in stroke. ROSIER and FAST-ED demonstrated intermediate accuracy profiles. Importantly, all four scales showed good discriminatory ability, with comparable overall performance, suggesting that several of these tools may be suitable for use in routine emergency stroke triage.

The ROSIER scale had good sensitivity (86.8%) but the lowest specificity (58.1%). This is most probably because it includes symptoms like seizures and altered consciousness—features that are frequently present in stroke mimics. Previous studies have cited a broad range of specificity for ROSIER (44%–86%), depending on the clinical setting and examiner experience [16, 18, 19]. Despite this shortcoming, the scale’s moderate AUC (0.813) justifies its use as a general screening tool, especially in the emergency department, where the early recognition of possible stroke cases is critical. Notably, its performance in the hands of trained EMS or ED staff seems to be enhanced, emphasizing the need for standardized application and context-driven interpretation [20].

The CPSS showed excellent sensitivity (90.7%) and the most significant AUC (0.860) among the instruments studied, thus confirming its strong potential for AIS detection in emergency settings. Its low NLR (0.14) also supports its effectiveness in ruling out stroke cases, making it a valuable triage tool, especially in prehospital settings. However, its specificity had wide variation—ranging from a low of 17% to a high of 96% in other studies—suggesting that the CPSS may yield false-positive outcomes, particularly in settings where the prevalence of stroke mimickers is high [8, 21, 22]. This variation underscores the need for context-specific application. At the same time, the CPSS is ideal for accelerated detection; its modest specificity (67.7% in this study) should be considered in resource-poor settings, where over-triage and unnecessary imaging can overcome emergency care systems [21, 22].

NIHSS had the highest specificity (81.48%) and a good AUC of 0.822, indicating that it is the most reliable tool to confirm stroke diagnosis in cases where there is clinical suspicion. The PLR (3.97) was also the highest of all the tools, implying that a positive NIHSS test result strongly raises the post-test probability of AIS. These results are in accordance with research like Heldner et al., which proved that NIHSS scores ≥ 8 are strongly associated with greater stroke severity and worse neurological deficit at presentation [23]. NIHSS, however, needs to be administered by trained clinicians, which limits its use in prehospital triage. Its complexity and time requirements, though warranted in the hospital setting, may decrease its feasibility for quick screening outside the tertiary care setting [24].

In our analysis, the FAST-ED scale had a balanced diagnostic profile with 74.7% sensitivity, 77.4% specificity, and 0.811 AUC, indicating good performance for the detection of AIS. Although its sensitivity was inferior to CPSS and its specificity was inferior to NIHSS, its overall accuracy and moderate PLR (3.31) and NLR (0.33) are suggestive of clinical usefulness, particularly in the prehospital setting. Previous reports have described comparable performance with sensitivities ranging from 60 to 77% and specificities ranging from 65 to 89%, but also describe a significant false-negative rate (22.5–28.8%), with concern for missed diagnoses—especially in non-cortical strokes [7, 25]. As FAST-ED places an emphasis on cortical signs, including eye deviation and neglect, its sensitivity would be expected to fall in patients without these symptoms [26]. Nevertheless, FAST-ED remains a practical bedside tool for rapid triage where early identification of suspected stroke and timely referral are priorities [27, 28].

Our analysis demonstrated no statistically significant differences in diagnostic performance (AUROC) between the four stroke assessment tools—NIHSS, CPSS, FAST-ED, and ROSIER—according to DeLong’s test. This is in line with a number of recent studies, which concluded that although each tool has special clinical strengths, none is universally superior in every environment. For example, Antipova et al. (2019) and Chaudhary et al. (2022) stated that tools like NIHSS, FAST-ED, and the Rapid Arterial Occlusion Evaluation (RACE) provide excellent diagnostic accuracy for LVO detection, in large part due to their inclusion of both cortical and motor function evaluation, but stressed that overall performance is still context-dependent [26, 29]; however, the RACE scale was not considered in our study, which was focused on tools most commonly applied and validated in both prehospital and EDs. Nevertheless, future research should involve RACE, the Los Angeles Motor Scale (LAMS), and other novel tools in order to provide a more extensive and inclusive analysis, especially given changing stroke triage protocols. Similarly, CPSS has been widely noted for high sensitivity in prehospital settings, as identified in the reviews by Rudd et al. (2015) and Baratloo et al. (2022). However, its specificity is typically lower than that of more extended tools such as NIHSS [30, 31]. Meanwhile, the ROSIER scale, as identified by Rad et al. (2023), demonstrates consistently high sensitivity (approximately 89–91%) but variable specificity, rendering it better suited to rapid screening rather than definitive diagnosis [12]. These comparative observations support the view that although there are minor numerical differences in AUC values and sensitivity/specificity, these differences fail to achieve statistical significance and are unlikely to change clinical decision-making in isolation. Thus, the selection of a stroke assessment tool must be individualized to the particular clinical environment—prehospital vs. in-hospital, for example—and weighed against practical considerations of time, examiner expertise, and the probability of stroke mimics within the population being screened.

In addition to diagnostic measures, patient demographics and patterns of clinical presentation also influenced tool performance. Stroke-diagnosed patients were considerably older, consistent with the well-established contribution of aging to stroke risk [32]. Stroke patients were also more likely to be male, which could have implications for symptom presentation and the sensitivity of tools that emphasize characteristic motor features [33, 34].

A critical contextual factor was the substantially shorter duration from symptom onset to ED presentation for stroke patients compared with non-stroke patients. Earlier presentation likely enabled more accurate identification of neurological deficits and may have enhanced the apparent diagnostic performance of the scales at triage. In contrast, delayed presentation among non-stroke patients may be related to evolving, fluctuating, or subtle symptoms, increasing diagnostic uncertainty and potentially lowering specificity. Time from symptom onset to ED arrival therefore represents a plausible confounder of scale performance. Because the study was not designed or powered to formally adjust for this variable, we were unable to statistically isolate its effect, and our results should be interpreted in light of this limitation [35, 36].

Strengths of this study are the prospective design, consecutive sampling, blinded neuroimaging review, and standardized application of four common stroke assessment instruments. The use of MRI as a reference test and robust statistical comparisons (e.g., DeLong’s test) increases the validity and reliability of the results. This study has several limitations. First, we evaluated the diagnostic performance of the scales for AIS overall and did not perform a dedicated, imaging-based subgroup analysis for LVO. Second, we did not formally assess the inter-rater reliability of the clinical scores, which could affect the reproducibility of these tools across different clinicians. Third, other validated prehospital instruments, such as RACE and LAMS, were not included in our comparison. Furthermore, the single-center design and strict inclusion criteria yielded a highly selected population, limiting the external validity and generalizability of our findings to broader cohorts, less-resourced, or prehospital environments. Additionally, unadjusted differences in symptom onset-to-presentation times and the heterogeneity of stroke mimics may have introduced residual confounding. Future multicenter studies in diverse settings, incorporating explicit control for key confounders and newer tools like AI-based decision-support systems, are warranted to robustly define the most effective triage strategies.

## Conclusion

All four stroke assessment tools (CPSS, FAST-ED, ROSIER, NIHSS) showed high diagnostic accuracy. CPSS was the most sensitive and suitable for early detection in prehospital care, while NIHSS had the highest specificity, making it valuable for diagnosis confirmation and severity assessment in hospitals. Despite no significant differences in overall effectiveness, each tool offers distinct advantages depending on context, so stroke triage should be adapted to the setting, resources, and staff expertise.

## Data Availability

The data sets utilized and analyzed in the present study are available upon reasonable request from the corresponding author.

## Declarations

### Generative AI and AI-assisted tools declaration

Generative AI tools were utilized to enhance the grammar and clarity of the manuscript text. The authors affirm that all intellectual content, study design, data analysis, and interpretation were conducted by the research team, and responsibility for the accuracy and integrity of the manuscript lies solely with the authors.

## Abbreviations

AUC: Area under the curve
PPV: Positive predictive value
NPV: Negative predictive value
PLR: Positive likelihood ratio
NLR: Negative likelihood ratio
CI: Confidence interval
